# The future of target-controlled infusion and new pharmacokinetic models

**DOI:** 10.1097/ACO.0000000000001529

**Published:** 2025-05-26

**Authors:** Anthony R. Absalom, Thomas W. Schnider

**Affiliations:** a Department of Anesthesiology, University Medical Center Groningen, University of Groningen, Groningen, The Netherlands; b Kantonsspital St. Gallen, St. Gallen, Switzerland

**Keywords:** pharmacokinetic and pharmacodynamic models, propofol, remifentanil, target, controlled infusion, total intravenous anesthesia

## Abstract

**Purpose of review:**

To summarize recent developments in the understanding of the pharmacology of the hypnotic and opioid drugs, with relevance to target-controlled infusions and newer pharmacokinetic models.

**Recent findings:**

General-purpose models have been developed for propofol, remifentanil, and dexmedetomidine, suitable for use in a wide variety of patients, but still not universally applicable. A validation study of the predictive performance of the Eleveld propofol model showed reasonable performance in children, healthy adults, and obese adults but poorer performance in elderly patients. Observational studies show that complications during total intravenous anesthesia often arise from omission of basic safety checks and inadequate knowledge, rather than model misspecification. Specifically, there is a lack of understanding of the influence of the clinical situation on the pharmacodynamics of hypnotic drugs. Artificial intelligence is likely to produce useful drug infusion rate advisory systems, or even closed-loop control systems that could potentially provide better patient-individualized titration of anesthetic drugs.

**Summary:**

Further efforts to develop new models are unlikely to be clinically beneficial. Efforts should rather be made to ensure better education and a better appreciation of variability in pharmacodynamics and the need for better ways of tailoring drug doses to individual patient needs.

**KEY POINTS**

- Increasing sophistication of pharmacokinetic models for propofol and remifentanil is unlikely to produce more accurate or more universally applicable models.
- Only a small portion of residual pharmacokinetic variability is likely to be explainable by patient characteristics, and this variability is much smaller than the interindividual variability in pharmacodynamics.
- Better, safer patient care will be better served by efforts to ensure basic safety steps are followed, and to ensure better appreciation of pharmacodynamic variability, and better patient-individualized drug titration

## INTRODUCTION

Since the commercial launch of target-controlled infusion (TCI) pumps, the technology has been used around much of the world [1], and in recent years, there has been a surge in clinical use of intravenous anesthesia techniques. Nationwide UK activity surveys have shown that the proportion of patients receiving total intravenous anesthesia (TIVA) increased from 8% in 2013 [2] to 27% in 2021 [3]. This has been driven by several factors, including the potential reduction in the incidence of postoperative nausea and neurocognitive disorders, and the limited environmental impact [4,5^■^,6^■^].

There have been many new developments in technology and in our understanding of the pharmacokinetics and pharmacodynamics of intravenous anesthetic drugs. The first generation of TCI pumps contained inbuilt safety systems such as a syringe recognition system, requiring the use of prefilled propofol syringes. The omission of such safety systems from subsequent generations of TCI pumps – the so-called ‘open-TCI’ pumps – was a step forward as it enabled application of TCI with other drugs and drug concentrations, but it can also be seen as a retrograde step, as it increased the risk of drug errors. Safety remains an important topic, as has the accuracy of the pharmacokinetic and pharmacodynamic (PKPD) models. As TCI technology is used for a significant portion of TIVA (about 70% in the UK [2]), and TIVA use is likely to grow further, it is apposite to once again review the current status and future of TCI, as well as that of the underlying pharmacokinetic and pharmacodynamic models.

## SAFETY GUIDELINES

TCI systems are safe in the sense that the equipment does what it is programmed to do in a reliable and accurate way [7]. The ultimate safety of the patient depends, however on how the anesthesiologist uses the equipment.

A large UK-based observational study of accidental awareness during anesthesia showed that the incidence of awareness was higher during intravenous than inhalational anesthesia [8]. A report containing detailed case analyses of TIVA-related awareness showed that in many cases inadequate knowledge and experience contributed significantly, and several cases were preventable [9]. Following the report, an expert working party developed guidelines for safe TIVA administration. They considered that all anesthesiologists should be proficient in TIVA, because all will encounter situations where volatile anesthesia is undesirable or contraindicated. Moreover, the guidelines recommended that given the complex pharmacokinetics of modern anesthetic drugs, TCI should be the default for TIVA administration [10^■^], implying that this technology can assist and simplify TIVA administration, potentially improving safety, especially for newer users. Importantly, these guidelines emphasize the prime importance of basic safety steps, such as ensuring that the correct drug and model are being used and that infusion lines and cannulae are visible and safely connected. In terms of choice of pharmacokinetic model, the guideline stressed that knowledge of and experience with any one model is more important than the choice of model.

Since then, another large observational study investigated perioperative cardiac arrests and found that the incidence of cardiac arrest was higher with TIVA than with volatile anesthesia [11^■■^]. Detailed analyses were performed of the cardiac arrest cases during which TIVA was thought to have played a role, which included cases during which TCI technology was used [12]. Risk factors for perioperative cardiac arrest were older age, higher ASA grade, and frailty. Prearrest care was judged poor in more than 50% of TIVA-related cardiac arrest cases. Inadequate knowledge was again highlighted, including poor understanding of important pharmacodynamics issues and ‘failure to tailor’ doses or initial target concentrations to the patient and clinical situation. Intermittent bolus doses or manual infusions instead of TCI administration was also considered to have contributed in some cases.

Since then, Japanese guidelines for safe TIVA practice have also been published [13^■^]. In common with the UK guidelines, they emphasize the importance of basic knowledge and practical safety steps. Both guidelines stress the importance of depth of anesthesia monitoring, especially when muscle relaxants are used. Both stress the limitations of current processed electroencephalogram (pEEG) monitors, and the importance of evaluation of signal quality and the unprocessed waveforms, in combination with all other clinical information [10^■^,13^■^].

These limitations of current pEEG systems have been broadly recognized, with recent work emphasizing less reliance on the indices shown by the monitors and more reliance on the raw EEG waveforms and on the frequency spectrogram when shown [14^■^,15^■■^]. These limitations of pEEG devices are relevant to TIVA practice in several ways, as pEEG indices have been used to estimate important pharmacodynamic components (e.g. ke0) of models such as the Eleveld propofol model [16].

## MIXING

Real-life practice commonly differs from ideal standards such as the advice of guidelines [10^■^] and editorials [17,18] not to mix drugs for infusion. In practise a mixture of remifentanil (a water soluble drug) and propofol (a lipophilic drug formulated in a fat emulsion) is commonly used, and in recent years, authors from several busy and reputable pediatric and adult centers have presented evidence of a lack of harm and a low incidence of complications with this technique [19–22,23^■^].

One of the arguments against the use of mixing of propofol and remifentanil is the fact that the two drugs have very different pharmacokinetic profiles [17,18]. Users of a mixture either infuse it by TCI from a pump programmed with the pharmacokinetics of propofol [20], or by manually controlled mass-based infusions [23^■^], in both cases titrated primarily on the basis of hypnotic endpoints. When TCI is used, changes in the target propofol concentration are likely to lead to large swings in the plasma remifentanil concentration, with the extent depending on the infusion rates and the concentration of remifentanil in the syringe (pediatric groups tend to use mixes with nominal concentrations of 5 mcg/ml remifentanil in 10 mg/ml propofol, whereas adult groups have reported a mix of 20 mcg/ml remifentanil in 10 mg/ml propofol).

During an optimally tailored anesthetic, the required ratio between the hypnotic and opioid effect-site concentrations can change. With a mixture, regardless of how it is administered, when infusion rates are reasonably stable, the ratio of plasma and effect-site propofol and remifentanil concentrations will be a fixed ratio, meaning that subtle control of the hypnotic and antinociceptive components of anesthesia is impossible.

These groups have evaluated the incidence of critical incidents, and found them to be low, and an adult group has shown that anesthesiologists using the mix in healthy young orthopedic patients were able to provide remarkably stable anesthesia in terms of depth of anesthesia, heart rate, and arterial blood pressure [23^■^]. In fact, the stability that they reported was somewhat better than that of a service evaluation in a setting when separate TCI-based infusions of propofol and remifentanil were used [24]. While we too advise against mixing, the stability of anesthesia achieved with it strengthens our conviction that TCI systems should be seen as a tool to assist titration of drug administration, as a gain switch [25^■^], but not as the ‘holy grail’. In the final analysis, the choice of model used is less important than the knowledge of the user, the clinical intuition and experience of the user gained through adequate training and case exposure, and close patient observation.

## NEW GENERAL-PURPOSE MODELS

A wide range of pharmacokinetics and PKPD models for different drugs and patient groups have been published and implemented in the ‘open TCI’ pumps introduced since 2003. Enlund [26] wisely pointed out that this was not only confusing for anesthesiologists, it also introduced safety risks, such as the risks of mistakenly using a model for the wrong drug, patient, or group. This motivated the development of general-purpose PKPD models, which ideally would be suitable for use in all patients for TCI administration of a specific drug. The subsequent development and validation of general-purpose models for propofol, remifentanil, and dexmedetomidine have been described and discussed in detail [27–29].

In brief, the Eleveld remifentanil model was developed using data from 131 mostly Caucasian patients, whose ages ranged from 0 to 85 years, and weights from 2.5 to 106 kg [30]. While this model is suitable for use in children, adults, and even octogenarians, it is not suitable for use in very obese children or adults. The Kim model, on the other hand, is suitable for use in healthy adults and adults with obesity, but not in children [31]. It was developed using data from 229 adult patients with ages from 20 to 85 years and weights from 45 to 215 kg.

The Eleveld PKPD model for propofol was developed using data from 1033 patients, with an age range from 0 to 88 years, and a weight from less than 1 to 160 kg [16]. It uses allometric scaling to calculate clearances, which may more accurately scale for size, but while it is suitable for use in overweight patients, it should probably not be used in patients with very severe obesity (i.e. a BMI > 40), who are increasingly presenting for surgery. Some pump manufacturers implement a maximum weight of 160 kg.

Of these general-purpose models, only the Eleveld propofol model has been prospectively validated [32]. Four groups of patients were studied – children, healthy adults, obese adults, and elderly adults – and the Varvel criteria were used to assess the predictive performance of the Eleveld model as well as that of other models. The criteria used were median performance error (a measure of bias), median absolute performance error (MDAPE, a measure of precision), and wobble (a measure of variability around the MDAPE) [33]. In terms of bias and precision, in adults and obese adults the Eleveld model performance was equivalent to that of the existing general adult models (Marsh *et al.* [34] and Schnider *et al.* [35,36]) and a model specific for obese adults (Cortinez [37]). For children the bias and precision were equivalent to that of the Paedfusor model [38], whereas in elderly patients the bias and precision were worse than that of the Marsh and Schnider models.

A unique feature of the Eleveld propofol model is that it incorporates the pharmacodynamic parameters EC50 and gamma (slope) alongside with ke0, developed from the data of studies involving bispectral index (BIS, Medtronic, Dublin, Ireland) recordings [16]. The ke0 is essential for effect-site targeting mode, and significantly impacts the size of loading/bolus dose during effect-site targeted TCI. The EC50, along with the slope parameter, can help the user select an age-appropriate starting target concentration. These parameters can also be used to predict the BIS for any given patient and effect-site concentration, but as mentioned, the BIS is an imperfect measure of anesthetic depth, particularly during the onset of effect, which might impair the accuracy of effect-site targeting (i.e. the degree of plasma/effect-site over- and undershoot) and of the BIS predictions.

Coetzee *et al.* [39] compared measured BIS values with the predictions of the Eleveld model. Their data show pharmacodynamic variability – target concentrations between 2 and 6 mcg/ml were required for adequate anesthesia – of similar order to that shown previously. Their data also showed that the BIS predictions were inaccurate. Bland–Altman analysis showed that although the mean bias was small, the limits of agreement were wide (−27.7 to 26.2 BIS units), and the authors concluded that the BIS predictions cannot replace actual BIS or other pEEG measurements.

These findings support the belief of many experts that increasing model sophistication does not necessarily produce better performance. The main incremental benefit of general-purpose models is that they have the potential to reduce drug errors, if TCI pumps are programmed with one general-purpose model for each drug. Unfortunately, these models cannot be considered ‘universally’ applicable and cannot replace all other models.

It seems unlikely that universal models will ever be feasible. Some years ago, Schnider *et al.* [36] studied the pharmacokinetics of propofol in age-stratified (range: 25–81 years) healthy volunteers who received propofol on two occasions, 1 week apart. Interestingly, during model development, when comparing a model with covariates, that is, adjustments for the volunteer characteristics – to a model without covariates, the inaccuracy only increased modestly, from 24 to 17.4%. Further, their data showed large intraindividual variability, of a similar magnitude to the interindividual variability.

Clinicians know that during induction of anesthesia in anxious patients with high heart rates and cardiac outputs, the pharmacokinetics of propofol will change after induction, when sympathetic activity, heart rate, and cardiac output have declined. There are a vast number of factors, some known, many unknown, that result in within- and between-subject variability in pharmacokinetics. Future implementation of corrections for all these factors in a pharmacokinetic or PKPD model is not feasible, particularly when these variables change during the course of an anesthetic.

## ARTIFICIAL INTELLIGENCE

Computer technology and more recently artificial intelligence have been increasingly applied in medicine, and have the potential to significantly improve intravenous drug delivery, by refining current systems or the development of novel approaches.

Efforts have been made to investigate whether artificial intelligence, and in particular machine learning, can assist with the development of more accurate pharmacokinetic models. Machine learning was applied to retrospectively acquired remifentanil data, and the results of traditional non-linear mixed effects modelling (NONMEM) analysis were compared with pharmacokinetic parameters developed by a support vector machine (SVM) and an artificial neural network (ANN) [40]. While the parameters from the ANN outperformed those from the NONMEM analysis, the best performance arose from the means of the NONMEM and ANN-generated parameters. The SVM-generated parameters produced poor accuracy.

As mentioned before, all pharmacokinetic models used for TCI are imperfect, and within a given patient, the pharmacokinetic processes of drug distribution and metabolism can change significantly over short periods of time [36]. Following the development of a point-of-care propofol measurement system, a Bayesian optimization algorithm was developed and tested [41]. Plasma propofol concentration measurements during the first hour of anesthesia were used to individualize volumes and clearances of a preliminary version of the Eleveld pharmacokinetic model. Performance was then assessed during the subsequent hour. Although bias improved, precision was worse, and control of depth of anesthesia and hemodynamic stability were unchanged. Further studies are needed to determine whether different algorithms or sampling schemes produce better results.

An alternative to live adaptation of the pharmacokinetic model is to provide anesthesiologists with real-time advice on the optimal choice of target concentrations. This is logical given the fact that pharmacodynamic variability is significantly greater than pharmacokinetic variability. A recently reported study randomized 100 patients with a wide range of ages and health status to two groups. In one group, the responsible anesthesiologist received real-time, continuous advice on the optimal choice of effect-site target concentration from a patient-individualized Bayesian pharmacodynamic advisory tool [42]. Availability of the information of the tool was not associated with an improvement in control of depth of anesthesia, or any of the secondary parameters.

Closed-loop control computer systems have been applied to anesthesia for several years [43]. Earlier systems used simpler proportional-integral-differential (PID) control algorithms to determine the target propofol concentrations of a TCI system, based on the error between the measured value and the setpoint of a pEEG index. These systems were not necessarily intelligent or able to learn, whereas systems that learn or are adaptive can be regarded as using artificial intelligence.

Schamberg *et al.* [44] developed a reinforcement learning paradigm that could be used as the control algorithm for a system that determines the target effect site propofol concentration for the Schnider model. When tested with simulated data it outperformed a PID algorithm, and when its performance was tested retrospectively on pEEG data acquired from patients, the outputs of the system rapidly converged with those of anesthesiologists.

In a groundbreaking study, Lee *et al.* [45] used deep learning to train an ANN system to predict BIS values based on propofol and remifentanil infusion rates. The predictive performance of the ANN was better than when propofol and remifentanil concentrations were estimated by the Schnider and Minto models, and a response surface pharmacodynamic model was used to estimate the BIS.

Yun *et al.* [46^■^] used a PKPD model to train a reinforcement learning system to learn the relationship between propofol infusion rate and a pEEG index, and proposed using the system to provide clinicians on the infusion rates needed for a target pEEG index value, in patients receiving remifentanil.

## THE DRUG TITRATION PARADOX

The performance of pharmacokinetic/pharmacodynamic models for anesthetic drugs is commonly evaluated using data from electronic patient records, or other databases containing the details of intraoperative drug administration data and measurements of pEEG indeces and other physiological parameters. At times, similar data have even been used to refine pharmacokinetic models for drugs, when the drugs were administered by TCI and blood samples were taken for plasma drug concentration analysis.

There are pitfalls to this approach, which came to light from an analysis of data from clinical practice involving more than 4500 patients [47]. In the hospital, propofol was administered by TCI according to an institutional best practice policy, with a target BIS of 40–60 [47]. Across patients, the central 95% of propofol target effect-site concentrations (CeT) after incision ranged from 1.5 to 3.5 µg/ml. Interestingly, when the authors searched for a possible systematic bias related to patient characteristics, almost none could be detected, meaning that only a very small fraction of this variability could be attributed to model imprecision, strengthening our belief that further refinements to pharmacokinetic and pharmacodynamic models are unlikely to yield significant performance improvements.

An incidental but significant and counter-intuitive observation was that higher propofol concentrations correlated with a lesser drug effect, a finding that challenges traditional pharmacological understanding. Further investigations using additional data confirmed this paradox, not only for propofol TCI with BIS as the titration target but also for volatile anesthetic end-tidal concentrations with BIS monitoring and norepinephrine infusion rates with mean arterial pressure as the target [48].

Schamberg and Brown [49] suggested that the drug titration paradox is a case of Simpson’s paradox, which can be summarized as a paradox observed when the slope of the correlation between two variables is inverted in the absence or presence of a third conditioning variable. In the case of the drug titration paradox, titration changes the relationship between dose and effect from a positive to a negative correlation. Shafer and Stanski [50] used simpler terminology in their commentary on this finding, by stating that titration turns pharmacology ‘upside down’. Titration changes the correlation between dose and effect, because without it, the dose is the independent, and the effect the dependent variable, whereas with titration, the clinical effect will determine the choice of dose making the effect the independent variable.

In theory, if all confounding variables and causal drug–effect relationships were properly accounted for in statistical modeling – for example, by stratification as argued by Schamberg – it might be possible to resolve this paradox [49]; however, even with sophisticated mixed-effects models that can account for interindividual variability, unknown confounders still impact the observed effect-to-dose relationship.

One of the clinically relevant confounders is the variability in stimulation intensity, which cannot be reasonably incorporated into a pharmacokinetic/pharmacodynamic model. Such confounders are likely the reason for the observation that the drug titration paradox also occurs in individual titration data [51^■■^].

Traditional dosing strategies establish a direct dose-to-effect relationship, whereas titration to a target effect follows an effect-to-dose relationship, where the administered dose is dependent on the observed effect. This fundamental distinction has major implications for clinical pharmacology. One crucial implication is that clinical drug titration data, which are inherently based on an effect-to-dose relationship, must not be used to model the dose-to-effect relationship. This is particularly relevant for the observed negative correlation between target propofol concentrations and BIS values, which indicates that titration was incomplete. If titration were perfect, the observed effect would be identical for all patients, and the spread of target concentrations would be even broader than what was observed in the clinical dataset analyzed by Schnider *et al*. [47].

This further supports the conclusion that improvements in pharmacokinetic model predictions will have limited clinical utility given the wide range of target concentrations and the inherently unpredictable individual patient requirements. Consequently, the prediction of maintenance concentrations of anesthetics remains a futile endeavor. Instead, the true clinical value of TCI lies in its ability to facilitate the identification of the individually appropriate concentration through continuous titration, rather than in the precision of initial model-based concentration predictions [25^■^].

## CONCLUSION

While TCI is generally safe, better education of anesthesiologists is needed, with a focus on basic safety measures and basic pharmacokinetic and pharmacodynamic knowledge. General purpose pharmacokinetic/pharmacodynamic models have been developed, and may help to make TCI administration safer if pumps can be programmed with fewer models. Further refinements to current models are unlikely to generate more accurate models and are unlikely to result in universally suitable models. Future research should focus on better methods of assessing clinical effect and on more accurate titration. Artificial intelligence may assist with this, and is likely to involve model-free advisory or even closed-loop control systems. Researchers should be aware of the drug titration paradox and design studies appropriately by choosing methods involving fixed doses to enable accurate quantification of the dose–response relationship.

## Acknowledgements

*None.*

## Financial support and sponsorship

*None.*

## Conflicts of interest

A.R.A. has received honoraria from Paion (Aachen, Germany) and Philips (Eindhoven, The Netherlands) – for advice – and has performed investigator-initiated research for Philips (The Netherlands). He is a member of the editorial board of the *British Journal of Anesthesia* and a financial trustee of the BJA Company. T. S. reports a financial relationship with Codan Argus (Baar, Switzerland) and Medcaptain Europe B.V. (Andelst, The Netherlands; consultancy).

## References

[1] Absalom AR Glen JB Zwart GJC. Target-controlled infusion: a mature technology. Anesth Analg 2016; 122:70–78. 26516798 10.1213/ANE.0000000000001009

[2] Sury MR Palmer JH Cook TM Pandit JJ. The state of UK Dental Anaesthesia: results from the NAP5 Activity Survey. A national survey by the 5th National Audit Project of the Royal College of Anaesthetists and the Association of Anaesthetists of Great Britain and Ireland. SAAD Dig 2016; 32:34–36. 27145558

[3] Kane AD Soar J Armstrong RA.; Collaborators. Patient characteristics, anaesthetic workload and techniques in the UK: an analysis from the 7th National Audit Project (NAP7) activity survey. Anaesthesia 2023; 78:701–711. 36857758 10.1111/anae.15989

[4] Ramirez MF Gan TJ. Total intravenous anesthesia versus inhalation anesthesia: how do outcomes compare? Curr Opin Anaesthesiol 2023; 36:399–406. 37338939 10.1097/ACO.0000000000001274

[5■] Kampman JM Sperna Weiland NH. Anaesthesia and environment: impact of a green anaesthesia on economics. Curr Opin Anaesthesiol 2023; 36:188–195. 36700462 10.1097/ACO.0000000000001243 PMC9973446

[6■] Lawin-O’Brien R Schwartz ES Montgomery H. The climate crisis – actions to prioritize for anaesthesiologists. Curr Opin Anaesthesiol 2025; 38:9–16. 39526681 10.1097/ACO.0000000000001444

[7] Schnider TW Minto CF Struys MM Absalom AR. The safety of target-controlled infusions. Anesth Analg 2016; 122:79–85. 26516801 10.1213/ANE.0000000000001005

[8] Pandit JJ Andrade J Bogod DG; Royal College of Anaesthetists. 5th National Audit Project (NAP5) on accidental awareness during general anaesthesia: summary of main findings and risk factors. Br J Anaesth 2014; 113:549–559. 25204697 10.1093/bja/aeu313

[9] Nimmo AF Cook TM. Total intravenous anaesthesia. In: Pandit JJ Cook TM, editors. Accidentanil awareness during general anaesthesia in the United Kingdom and Ireland. Royal College of Anaesthetists, Association of Anaesthetists of Great Britain and Ireland; 2014. pp. 151–158.

[10■] Nimmo AF Absalom AR Bagshaw O. Guidelines for the safe practice of total intravenous anaesthesia (TIVA): joint guidelines from the Association of Anaesthetists and the Society for intravenous anaesthesia. Anaesthesia 2019; 74:211–224. 30378102 10.1111/anae.14428

[11■■] Kane AD Cook TM Armstrong RA; Collaborators. The incidence of potentially serious complications during non-obstetric anaesthetic practice in the United Kingdom: an analysis from the 7th National Audit Project (NAP7) activity survey. Anaesthesia 2024; 79:43–53. 37944508 10.1111/anae.16155

[12] Armstrong R Plaat F. Perioperative cardiac arrest and anaesthetic drug choice and dosing. Report and findings of the 7th National Audit Project. Royal College of Anaesthetists; 2023. pp. 256–262.

[13■] Obara S Kamata K Nakao M. Recommendation for the practice of total intravenous anesthesia. J Anesth 2024; 38:738–746. 39217587 10.1007/s00540-024-03398-2

[14■] Kreuzer M Garcia PS Gutierrez R Purdon PL. For EEG-guided anesthesia, we have to go beyond the index. Anesth Analg 2024; 139:e21–e22. 38885144 10.1213/ANE.0000000000007098

[15■■] Hight D Kreuzer M Ugen G. Five commercial ‘depth of anaesthesia’ monitors provide discordant clinical recommendations in response to identical emergence-like EEG signals. Br J Anaesth 2023; 130:536–545. 36894408 10.1016/j.bja.2022.12.026

[16] Eleveld DJ Colin P Absalom AR Struys M. Pharmacokinetic-pharmacodynamic model for propofol for broad application in anaesthesia and sedation. Br J Anaesth 2018; 121:519–559. 29661412 10.1016/j.bja.2018.01.018

[17] Absalom AR Rigby-Jones AE Rushton AR Sneyd JR. De-mystifying the ‘Mixifusor’. Paediatr Anaesth 2020; 30:1292–1298. 33051933 10.1111/pan.14039 PMC7756545

[18] Absalom AR Sneyd JR. Cutting corners – mixing remifentanil and propofol is a bad idea. Anesth Analg 2025 (in press). 10.1213/ANE.0000000000007599 40768374

[19] Bagshaw O. Pediatric anesthesia editorial-propofol and remifentanil: to mix or not to mix. Paediatr Anaesth 2016; 26:677–679. 27277649 10.1111/pan.12932

[20] Bagshaw O McCormack J Brooks P. The safety profile and effectiveness of propofol-remifentanil mixtures for total intravenous anesthesia in children. Paediatr Anaesth 2020; 30:1331–1339. 32961621 10.1111/pan.14018

[21] Malherbe S Barker N. Mixing of propofol and remifentanil. Paediatr Anaesth 2021; 31:504–505. 33772960 10.1111/pan.14137

[22] Allison E Malherbe S Barker N. Drug mixtures and infusion technology. Anaesthesia 2022; 77:835. 35187639 10.1111/anae.15694

[23■] Bennion NR Brower TS Ballard CR. Single syringe total intravenous anesthesia with propofol and remifentanil: a prospective cohort study. Anesth Analg 2025 (in press). 10.1213/ANE.0000000000007581 PMC12588638 40455662

[24] Gross T Feliot E Gayat E. Bispectral index during maintenance of total intravenous anesthesia: frequency of out of recommended range and impact of patients’ characteristics: a brief report. Anesth Analg 2020; 131:e52–e54. 31335404 10.1213/ANE.0000000000004313

[25■] Egan TD Minto CF Schnider TW. Steady-state trumps accuracy: target-controlled infusion as a gain switch. Br J Anaesth 2024; 133:726–729. 39304281 10.1016/j.bja.2024.07.014

[26] Enlund M. TCI: Target controlled infusion, or totally confused infusion? Call for an optimised population based pharmacokinetic model for propofol 6. Ups J Med Sci 2008; 113:161–170. 18509810 10.3109/2000-1967-222

[27] Vandemoortele O Hannivoort LN Vanhoorebeeck F. General purpose pharmacokinetic-pharmacodynamic models for target-controlled infusion of anaesthetic drugs: a narrative review. J Clin Med 2022; 11:2487. 35566617 10.3390/jcm11092487 PMC9101974

[28] Kuizenga MH Vereecke HE Struys MM. Model-based drug administration: current status of target-controlled infusion and closed-loop control. Curr Opin Anaesthesiol 2016; 29:475–481. 27152471 10.1097/ACO.0000000000000356

[29] Vellinga R Eleveld DJ Struys M van den Berg JP. General purpose models for intravenous anesthetics, the next generation for target-controlled infusion and total intravenous anesthesia? Curr Opin Anaesthesiol 2023; 36:602–607. 37678184 10.1097/ACO.0000000000001300

[30] Eleveld DJ Proost JH Vereecke H. An allometric model of remifentanil pharmacokinetics and pharmacodynamics. Anesthesiology 2017; 126:1005–1018. 28509794 10.1097/ALN.0000000000001634

[31] Kim TK Obara S Egan TD; The Remifentanil Pharmacokinetics in Obesity Investigators. Disposition of remifentanil in obesity: a new pharmacokinetic model incorporating the influence of body mass. Anesthesiology 2017; 126:1019–1032. 28509796 10.1097/ALN.0000000000001635

[32] Vellinga R Hannivoort LN Introna M. Prospective clinical validation of the Eleveld propofol pharmacokinetic-pharmacodynamic model in general anaesthesia. Br J Anaesth 2021; 126:386–394. 33317804 10.1016/j.bja.2020.10.027

[33] Varvel JR Donoho DL Shafer SL. Measuring the predictive performance of computer-controlled infusion pumps. J Pharmacokinet Biopharm 1992; 20:63–94. 1588504 10.1007/BF01143186

[34] Marsh B White M Morton N Kenny GN. Pharmacokinetic model driven infusion of propofol in children. Br J Anaesth 1991; 67:41–48. 1859758 10.1093/bja/67.1.41

[35] Schnider TW Minto CF Shafer SL. The influence of age on propofol pharmacodynamics. Anesthesiology 1999; 90:1502–1516. 10360845 10.1097/00000542-199906000-00003

[36] Schnider TW Minto CF Gambus PL. The influence of method of administration and covariates on the pharmacokinetics of propofol in adult volunteers. Anesthesiology 1998; 88:1170–1182. 9605675 10.1097/00000542-199805000-00006

[37] Cortinez LI Anderson BJ Penna A. Influence of obesity on propofol pharmacokinetics: derivation of a pharmacokinetic model. Br J Anaesth 2010; 105:448–456. 20710020 10.1093/bja/aeq195

[38] Absalom A Amutike D Lal A. Accuracy of the ‘Paedfusor’ in children undergoing cardiac surgery or catheterization. Br J Anaesth 2003; 91:507–513. 14504151 10.1093/bja/aeg220

[39] Coetzee E Coetzee JF Haasbroek M. Predictive pharmacodynamic performance of the Eleveld pharmacokinetic-pharmacodynamic model for propofol: comparison of predicted and measured bispectral index. Br J Anaesth 2024; 133:785–792. 39179443 10.1016/j.bja.2024.06.041

[40] Poynton MR Choi BM Kim YM. Machine learning methods applied to pharmacokinetic modelling of remifentanil in healthy volunteers: a multi-method comparison. J Int Med Res 2009; 37:1680–1691. 20146865 10.1177/147323000903700603

[41] van den Berg JP Eleveld DJ De Smet T. Influence of Bayesian optimization on the performance of propofol target-controlled infusion. Br J Anaesth 2017; 119:918–927. 29028925 10.1093/bja/aex243

[42] Vellinga R Introna M van Amsterdam K. Implementation of a Bayesian based advisory tool for target-controlled infusion of propofol using qCON as control variable. J Clin Monit Comput 2024; 38:519–529. 38112878 10.1007/s10877-023-01106-1

[43] Zaouter C Joosten A Rinehart J. Autonomous systems in anesthesia: where do we stand in 2020? A narrative review. Anesth Analg 2020; 130:1120–1132. 32287120 10.1213/ANE.0000000000004646

[44] Schamberg G Badgeley M Meschede-Krasa B. Continuous action deep reinforcement learning for propofol dosing during general anesthesia. Artif Intell Med 2022; 123:102227. 34998516 10.1016/j.artmed.2021.102227

[45] Lee HC Ryu HG Chung EJ Jung CW. Prediction of bispectral index during target-controlled infusion of propofol and remifentanil: a deep learning approach. Anesthesiology 2018; 128:492–501. 28953500 10.1097/ALN.0000000000001892

[46■] Yun WJ Shin M Jung S. Deep reinforcement learning-based propofol infusion control for anesthesia: a feasibility study with a 3000-subject dataset. Comput Biol Med 2023; 156:106739. 36889025 10.1016/j.compbiomed.2023.106739

[47] Schnider TW Minto CF Egan TD Filipovic M. Relationship between propofol target concentrations, bispectral index, and patient covariates during anesthesia. Anesth Analg 2021; 132:735–742. 32833715 10.1213/ANE.0000000000005125

[48] Schnider TW Minto CF Filipovic M. The drug titration paradox: correlation of more drug with less effect in clinical data. Clin Pharmacol Ther 2021; 110:401–408. 33426670 10.1002/cpt.2162 PMC8359232

[49] Schamberg G Brown EN. The drug titration paradox is Simpson’s paradox. Clin Pharmacol Ther 2021; 110:1424–1425. 34038588 10.1002/cpt.2280

[50] Shafer SL Stanski DR. The titration paradox turns pharmacology upside down. Clin Pharmacol Ther 2021; 110:292–293. 33852737 10.1002/cpt.2235

[51■■] Schnider TW Minto CF Luginbuhl M Egan TD. The drug titration paradox: more drug does not correlate with more effect in individual clinical data. Br J Anaesth 2022; 129:861–867. 35863951 10.1016/j.bja.2022.05.036

